# Similarity of eyes in a cataractous population—How reliable is the biometry of the fellow eye for lens power calculation?

**DOI:** 10.1371/journal.pone.0269709

**Published:** 2022-06-30

**Authors:** Achim Langenbucher, Nóra Szentmáry, Alan Cayless, Veronika Röggla, Christina Leydolt, Jascha Wendelstein, Peter Hoffmann

**Affiliations:** 1 Department of Experimental Ophthalmology, Saarland University, Homburg/Saar, Germany; 2 Dr. Rolf M. Schwiete Center for Limbal Stem Cell and Aniridia Research, Saarland University, Homburg/Saar, Germany; 3 Department of Ophthalmology, Semmelweis-University, Budapest, Hungary; 4 School of Physical Sciences, The Open University, Milton Keynes, United Kingdom; 5 Department of Optometry and Ophthalmology, AKH Vienna University Hospital, Vienna, Austria; 6 Department of Ophthalmology, Johannes Kepler University, Linz, Austria; 7 Augen- und Laserklinik Castrop-Rauxel, Castrop-Rauxel, Germany; Yenepoya Medical College, Yenepoya University, INDIA

## Abstract

**Background:**

In some situations it is necessary to use biometry from the fellow eye for lens power calculation prior to cataract surgery. The purpose of this study was to analyse the lateral differences in biometric measurements and their impact on the lens power calculation.

**Methods:**

The analysis was based on a large dataset of 19,472 measurements of 9736 patients prior to cataract surgery with complete biometric data of both left and right eyes extracted from the IOLMaster 700. After randomly indexing the left or right eye as primary (P) and secondary (S), the differences between S and P eye were recorded and analysed (Keratometry (RSEQ), total keratometry (TRSEQ) and back surface power (BRSEQ)), axial length AL, corneal thickness CCT, anterior chamber depth ACD, lens thickness LT). Lens power was calculated with the Castrop formula for all P and S eyes, and the refraction was predicted using both the P and S eye biometry for the lens power calculation.

**Results:**

Lateral differences (S-P, 90% confidence interval) ranged between -0.64 to 0.63 dpt / -0.67 to 0.66 dpt / -0.12 to 0.12 dpt for RSEQ / TRSEQ / BRSEQ. The respective difference in AL / CCT / ACD / LT ranged between -0.46 to 0.43 mm / -0.01 to 0.01 mm / -0.20 to 0.20 mm / -0.13 to 0.14 mm. The resulting difference in lens power and predicted refraction ranged between -2.02 to 2.00 dpt and -1.36 to 1.30 dpt where the biometry of the S eye is used instead of the P eye. The AL and RSEQ were identified as the most critical parameters where the biometry of the fellow eye is used.

**Conclusion:**

Despite a strong similarity of both eyes, intraocular lens power calculation with fellow eye biometry could yield different results for the lens power and finally for the predicted refraction. In 10% of cases, the lens power derived from the S eye deviates by 2 dpt or more, resulting in a refraction deviation of 1.36 dpt or more.

## Background

In some clinical situations biometric measurements of an eye prior to cataract surgery is difficult or even impossible using modern optical biometers. For example, severe opacifications in the lens or gas or silicone oil as replacement for the native vitreous body can prevent derivation of distances within the eye, and the only option is to use ultrasound techniques to measure relevant distances [1]. If proper fixation of the eye is lost, optical measurements performed under fixation generally fail [2]. As ultrasound biometry in general does not show the same accuracy as biometric measures [3–5], many surgeons today do not have sufficient skills to obtain reliable values. Furthermore, with ultrasound measurement being a contact or immersion measurement, it requires topical anaesthesia of the eye and therefore cannot be delegated to optometrists or health professionals. In general, this leaves the option of using biometric data from the fellow eye to calculate the appropriate lens power [1,6].

In general, both eyes of an individual are quite similar in axial length and corneal front surface radius of curvature, which is the minimum requirement for lens power calculations [7–10]. Even in myopic [11] or hyperopic patients [12] biometric parameters typically do not vary significantly between the two eyes. With modern formulae, additional parameters such as phakic anterior chamber depth, thickness of the crystalline lens [1], the horizontal corneal diameter, or the corneal back surface radius of curvature, together with the central corneal thickness [13] can be used to enhance the predictability of the refractive outcome after cataract surgery.

With the newest generation of optical biometers, all of these data are derived with a single on-axis measurement [4,5]. In contrast, older optical biometers (e.g. the IOLMaster 500, Carl-Zeiss-Meditec, Jena, Germany) did not assess some of the distances in the eye (e.g. crystalline lens thickness) [1,3]. Classical ultrasound biometers only give readings of distances within the eye and could not measure the corneal radius of curvature at all. This means that corneal curvature data would have to be extracted from a manual keratometer or a topographer, with the disadvantage that the axis of the ultrasound measurement does not necessarily coincide with the instrument axis of the keratometer or the topographer [14]. Additionally, the workflow with 2 separate measurements is more complex, as the measurement of corneal curvature has to be performed routinely before the ultrasound biometric measurement in immersion or contact [14], and for any potential subsequent optical measurement (e.g. refractometry) the patient would have to wait until the corneal shape and tear film is fully recovered.

The **purpose of the present study** was to record and analyse the differences between the left and right eye biometry in a large cataractous population using a modern optical biometer, and to predict the resulting intraocular lens power and refraction of the pseudophakic eye when biometric data from the fellow eye are used for lens power calculation.

## Methods

### Dataset for our analysis

In total, a dataset with 32,198 biometrical measurements from the IOLMaster 700 (Carl-Zeiss-Meditec, Jena, Germany) from two clinical centres (Augenklinik Castrop, Castrop-Rauxel, Germany and Department of Ophthalmology, Johannes Kepler University Linz, Austria) was considered for this retrospective study. Duplicate measurements of eyes, as well as incomplete data in the dataset were discarded. All measurements were performed in a cataractous population. Measurements from pseudophakic eyes or eyes in mydriasis and data indexed as after refractive surgery, ectatic corneal diseases such as keratoconus or keratoglobus, other corneal pathologies, or with ocular trauma were omitted from the dataset. The data were anonymised at the clinical centres and transferred to a.csv data table using the data export module of the IOLMaster 700 software. Data tables were reduced to the relevant parameters required for our data analysis, consisting of: laterality (left or right eye), patient’s date of birth and examination date of the eyes, curvature of the corneal front surface in the flat (R1) and the steep (R2) meridian both in mm including axis orientation of the flat meridian (RA), total keratometry TK as a composite value for the refraction of the cornea as thick lens expressed in radius of curvature in the flat (TR1) and the steep (TR2) meridian both in mm including axis orientation of the flat meridian (TRA), curvature of the corneal back surface in the flat (BR1) and the steep (BR2) meridian both in mm including axis orientation of the flat meridian (BRA), central corneal thickness (CCT in mm), anterior chamber depth (ACD) measured from the corneal front apex to the crystalline lens front apex in mm, central thickness of the crystalline lens (LT in mm), axial length (AL in mm), and the horizontal corneal diameter (W2W in mm).

The data were transferred to Matlab (Matlab 2019b, MathWorks, Natick, USA) for further processing. A waiver was provided for this study by the local ethics committee (Ärztekammer des Saarlandes, 157/21).

### Preprocessing of the data

Custom software for data processing and analysis was written in Matlab. Only patient records having valid measurements from both eyes were selected from the dataset. All other records were discarded. From the patient’s date of birth and the examination date we derived the age of the patients (Age in years). The curvature of the corneal front surface (R1, R2, and RA) was converted into keratometric power expressed in power vectors with RSEQ = 0.5 (332/R1+332/R2), RC0 = (332/R2-332/R1) cos(RA), RC45 = (332/R2-332/R1) sin(RA). The same conversion from curvature to power vectors was used for total keratometry (TRSEQ = 0.5 (332/TR1+332/TR2); TRC0 = (332/TR2-332/TR1) cos(TRA); TRC45 = (332/TR2-332/TR1) sin(TRA)) and corneal back surface curvature (BRSEQ = 0.5 (40/BR1+40/BR2); BRC0 = (40/BR1-40/BR2) cos(BRA); BRC45 = (40/BR1-40/BR2) sin(BRA)), respectively. To account for potential lateral symmetry of the power vectors, the power vector components for the oblique axis (RC45, TRC45 and BRC45) were flipped in sign for all left eyes to consider all eyes as right eyes (new variables: RC45OD, TRC45OD and BRC45OD).

The dataset was split into primary eyes (P) and secondary (S) eyes using a pseudo-random sequence. In each case, the primary eye was taken as the eye to be treated. The biometry and lens power calculations for both the P and the S eye from the same individual were then applied and the results compared. Lateral differences were documented for all parameters as the difference between the secondary eye and the primary eye measurement indexed by ‘S-P’.

### Intraocular lens power, predicting the refraction

The Castrop formula was used to calculate the intraocular lens power. This is a paraxial lens power calculation concept which uses a thick lens model for the cornea (mean corneal front and back surface curvature and central corneal thickness) and a thin lens model for the intraocular lens [13,15]. For the formula constants we used C = 0.34909, H = 0.33333, and R = -0.00275 as derived from the IOLCon WEB site (https://iolcon.org, accessed on 19.03.2022) for the ZCB00 lens (Johnson & Johnson Vision, Santa Ana, USA). These are based on a formula constant optimisation in 323 clinical results. Two of the 3 formula constants (C and H) act on the estimated axial lens position, whereas the third constant R accounts for a systematic shift in the refractive outcome. For the mean corneal front and back surface radius of curvature we used RF = 0.5 (R1+R2) and RB = 0.5 (BR1+BR2), respectively.

The lens power (IOLP) derived from the primary (IOLP_P_) and the secondary (IOLP_S_) eye biometry for emmetropia (target refraction zero) were also quantised in half dioptre steps (according to the normal delivery range) with IOLPq_P_ = 0.5 round(2 (IOLP_P_+0.15)) and IOLPq_S_ = 0.5 round(2 (IOLP_S_+0.15)) in such a way that lens powers were rounded asymmetrically with a lens power shift of 0.15 dioptre. In this context, "round(.)" refers to a numerical operation rounding to the nearest integer value.

The refraction at the spectacle plane (REF) in a vertex distance VD = 12 mm was calculated from the lens powers (REF_P_ from IOLP_P_ and REF_S_ from IOLP_S_), and in addition from the quantised intraocular lens powers (REFq_P_ from IOLPq_P_ and REFq_S_ from IOLPq_S_).

### Statistics

An explorative data analysis was performed using the arithmetic mean, standard deviation, median, as well as the lower and upper bounds of the 90% confidence intervals (5% and 95% quantiles) and the lower and upper bounds of the 99% confidence intervals (0.5% and 99.5% quantiles). The vector components of keratometry, total keratometry and corneal back surface power for the primary and secondary eyes are displayed in the Results section using boxplots. The vector differences of keratometry, total keratometry and corneal back surface power comparing the secondary and primary eyes are shown using 3D scatterplots. Differences in intraocular lens power and predicted refraction between secondary and primary eyes are provided in the form of cumulative density distribution (CDF) plots.

## Results

After quality approval of the dataset and filtering out incomplete data and records with measurements from only one eye, N = 19,472 measurements of (9736 right and 9736 left eyes from 9736 patients) were eventually used for our study. From the entire dataset, 9736 eyes (4858 left and 4858 right eyes) were indexed as P and 9736 eyes (4858 left and 4858 right eyes) as S.

The mean age of the study population was 69±15 years (median 72 years, 90% confidence interval from 43 to 85 years). The mean corneal curvature RF / RB of the corneal front / back surface was 7.71±0.27 mm, median 7.71 mm, 90% confidence interval 7.29 mm to 8.16 mm / 6.87±0.28 mm, median 0.28 mm, 90% confidence interval 6.43 mm to 7.34 mm. **Table 1** shows the explorative data for the power vector components for keratometry (RSEQ, RC0, RC45), total keratometry (TRSEQ, TRC0, TRC45) and corneal back surface power (BRSEQ, BRC0, BRC45) together with the distance measurements AL, CCT, ACD, and LT derived from the IOLMaster 700 biometer for the entire dataset. The respective values for RC45OD / TRC45OD / BRC45OD with the left eyes flipped horizontally to right eyes are -0.03±0.57 dpt (median -0.03 dpt, 90% confidence interval -0.85 to 0.83 dpt) / -0.05±0.59 dpt (median -0.06 dpt, 90% confidence interval -0.92 to 0.82 dpt) / -0.03±0.10 dpt (median -0.02 dpt, 90% confidence interval -0.19 to 0.13 dpt).

**Table 1:** Explorative data extracted from the dataset of the IOLMaster 700 biometer.

N = 19,472	RSEQ	RC0	RC45	TRSEQ	TRC0	TRC45	BRSEQ	BRC0	BRC45	AL	CCT	ACD	LT
	Keratometry in dpt			Total keratometry in dpt			Back surface in dpt			Distances in mm			
MEAN	43.11	0.20	-0.02	43.17	-0.01	-0.03	-5.83	-0.24	-0.01	23.68	0.55	3.13	4.61
SD	1.51	0.96	0.57	1.52	0.99	0.59	0.24	0.15	0.11	1.40	0.03	0.42	0.49
MEDIAN	43.08	0.15	-0.01	43.14	-0.03	-0.03	-5.83	-0.23	-0.01	23.48	0.55	3.13	4.64
5% quantile	40.68	-1.23	-0.86	40.75	-1.50	-0.91	-6.23	-0.49	-0.18	14.58	0.49	2.45	3.67
95% quantile	45.58	1.80	0.82	45.65	1.63	0.84	-5.46	0.0	0.15	37.54	0.61	3.84	5.34
0.5% quantile	39.22	-2.44	-1.99	39.22	-2.71	-2.07	-6.48	-0.73	-0.35	21.84	0.46	2.12	3.51
99.5% quantile	47.25	3.74	1.99	47.35	3.57	1.99	-5.24	0.18	0.31	26.14	0.65	4.21	5.77

RSEQ, RC0 and RC45 refer to the power vector components of keratometry expressed in equivalent power and projection of the astigmatism to the 0/90° and 45°/135° meridian, TRSEQ, TRC0 and TRC45 to the power vector components of total keratometry TK, and BRSEQ, BRC0 and BRC45 to the power vector components of the corneal back surface. MEAN, SD, and MEDIAN refer to the mean value, standard deviation and median value, and 5% / 9% quantiles and 0.5% / 99.5% quantiles to the 90% and 99% confidence interval, respectively.

**Fig 1:**
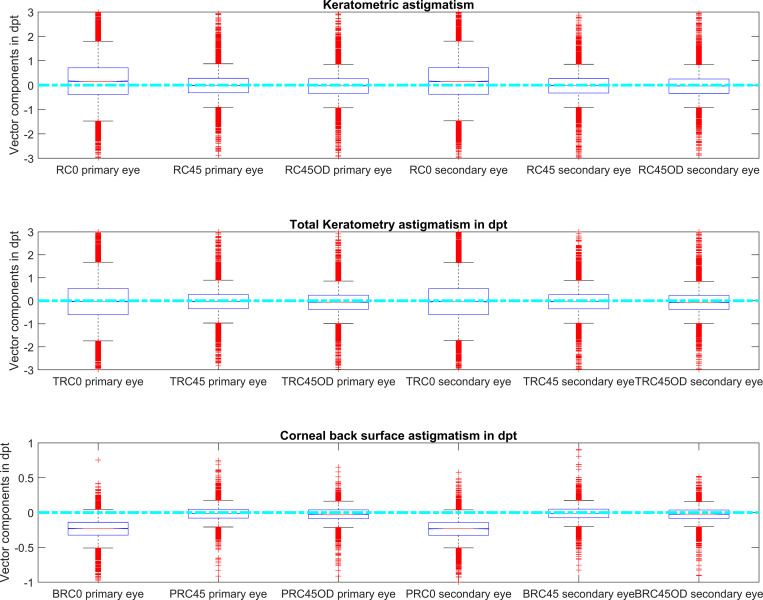
Boxplot of the power vector components for keratometry (upper graph), total keratometry (middle graph) and corneal posterior surface (lower graph). RSEQ, TRSEQ and BRSEQ refer to the equivalent power of keratometry, total keratometry and corneal back surface power, and RC0 / TRC0 / BRC0 and RC45 / TRC45 / BRC45 to the projection of the astigmatism to the 0° / 90° and the 45° / 135° meridians for keratometry / total keratometry / corneal back surface, respectively. RC45OD / TRC45OD / BRC45OD show the astigmatic vector components with RC45 / TRC45 / BRC45 for left eyes flipped in sign. The entire dataset is split randomly into primary eyes (to be treated with cataract surgery) and secondary eyes (whose biometry is used). The cyan line indicates stigmatic cases.

**Fig 1** displays the power vector components for keratometry, total keratometry and corneal back surface power together with the power components RC45OD, TRC45OD, and BRC45OD with the left eyes flipped horizontally to right eyes.

The difference in mean corneal curvature RF_S-P_ / RB_S-P_ of the corneal front / back surface comparing the secondary eyes and primary eyes was 0.52±81.40 μm, median 0.88 μm, 90% confidence interval -113.64 μm to 115.13 μm / 0.94±97.54 μm, median 0.82 μm, 90% confidence interval -136.80 μm to 137.06 μm. **Table 2** shows the explorative data for the difference (secondary eye minus primary eye) of the power vector components for keratometry (RSEQ_S-P_, RC0_S-P_, RC45_S-P_), total keratometry (TRSEQ_S-P_, TRC0_S-P_, TRC45_S-P_) and corneal back surface power (BRSEQ_S-P_, BRC0_S-P_, BRC45_S-P_) together with the differences in distance measurements AL_S-P_, CCT_S-P_, ACD_S-P_, and LT_S-P_ for the entire dataset. The respective values for RC45_S-P_OD / TRC45_S-P_OD / BRC45_S-P_OD with the left eyes flipped horizontally to right eyes are -0.01±0.60 dpt (median 0.00 dpt, 90% confidence interval -0.90 to 0.90 dpt) / -0.01±0.63 dpt (median 0.00 dpt, 90% confidence interval -0.96 to 0.93 dpt) / -0.00±0.12 dpt (median 0.00 dpt, 90% confidence interval -0.19 to 0.19 dpt).

**Table 2:** Explorative data of the difference between secondary and primary eye (S-P) extracted from the dataset of the IOLMaster 700 biometer.

N = 9736	RSEQ_S-P_	RC0_S-P_	RC45_S-P_	TRSEQ_S-P_	TRC0_S-P_	TRC45_S-P_	BRSEQ_S-P_	BRC0_S-P_	BRC45_S-P_	AL_S-P_	CCT_S-P_	ACD_S-P_	LT_S-P_
	Keratometry in dpt			Total keratometry in dpt			Back surface in dpt			Distances in mm			
MEAN	0.00	0.00	-0.01	0.00	0.00	-0.01	0.00	0.00	0.00	0.00	0.00	0.00	-0.02
SD	0.47	0.69	0.97	0.49	0.72	1.00	0.09	0.11	0.18	0.37	0.01	0.13	0.10
MEDIAN	0.00	0.00	-0.01	0.00	0.00	-0.01	0.00	0.00	0.00	0.00	0.00	0.00	0.00
5% quantile	-0.64	-1.03	-1.44	-0.67	-1.09	-1.49	-0.12	-0.18	-0.27	-0.46	-0.01	-0.20	-0.13
95% quantile	0.63	1.00	1.43	0.66	1.04	1.47	0.12	0.17	0.28	0.43	0.01	0.20	0.14
0.5% quantile	-1.45	-2.36	-3.42	-1.45	-2.44	-3.45	-0.21	-0.34	-0.55	-1.97	-0.03	-0.45	-0.33
99.5% quantile	1.43	2.29	3.35	1.53	2.34	3.44	0.22	0.35	0.54	1.81	0.03	0.446	0.32

RSEQ_S-P_, RC0_S-P_ and RC45_S-P_ refer to the power difference vector components of keratometry expressed in equivalent power and projection of the astigmatism to the 0/90° and 45°/135° meridian, TRSEQ_S-P_, TRC0_S-P_ and TRC45_S-P_ to the power difference vector components of total keratometry TK, and BRSEQ_S-P_, BRC0_S-P_ and BRC45_S-P_ to the power difference vector components of the corneal back surface. MEAN, SD, and MEDIAN refer to the mean value, standard deviation and median value, and 5% / 9% quantiles and 0.5% / 99.5% quantiles to the 90% and 99% confidence interval, respectively.

**Fig 2:**
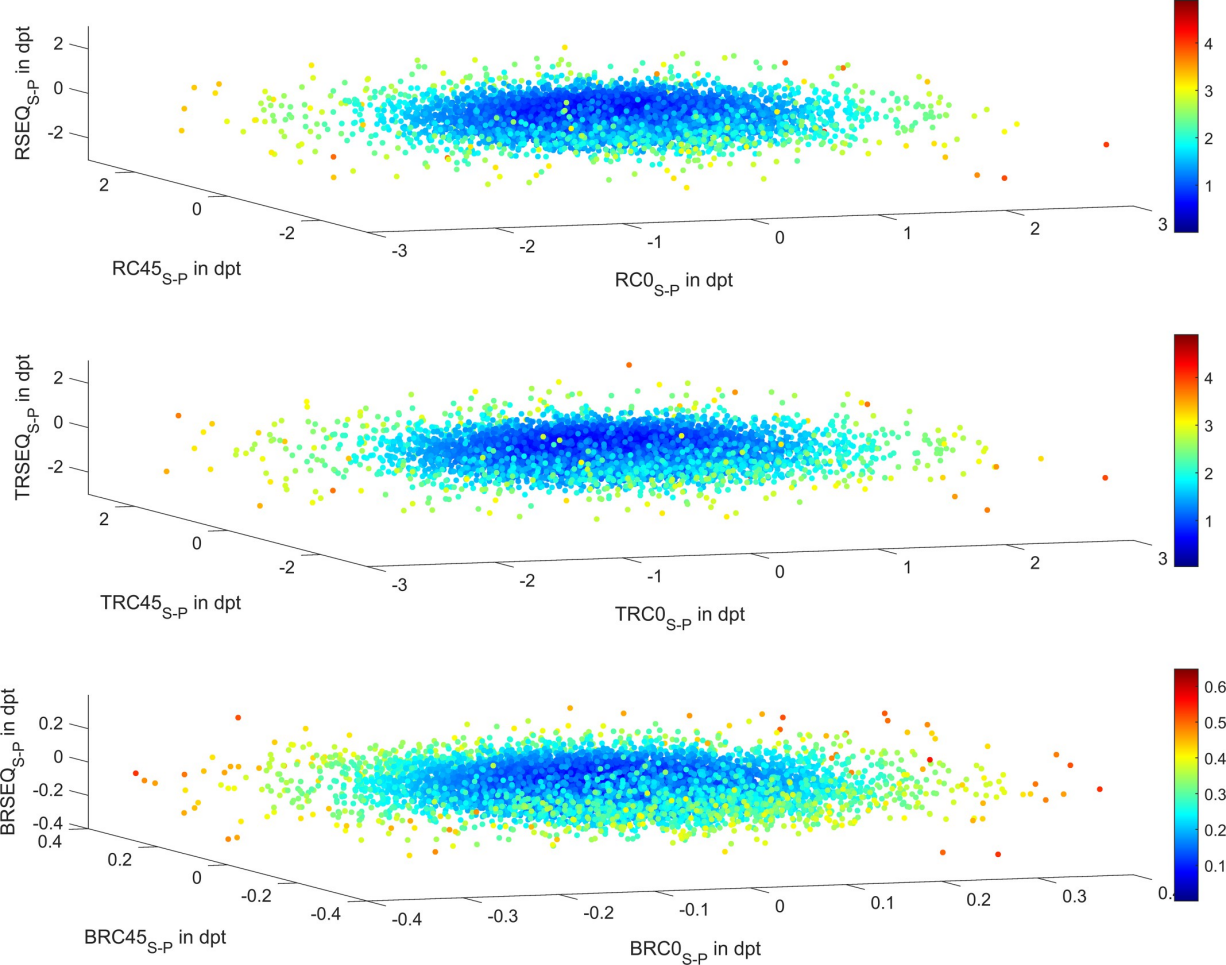
Difference of the power vector components comparing both eyes of the individuals (secondary eyes minus primary eyes) for keratometry (upper graph), total keratometry (middle graph), and corneal back surface (lower graph). The Euclidian norm of the power vector is colour-coded as indicated in the colour scale on the right.

**Fig 2** displays the scatterplot of differences in power vector components for keratometry (upper graph), total keratometry (middle graph) and corneal back surface power (lower graph). The plots are colour coded according to the Euclidian norm of the power vectors ((RSEQ_S-P_^2^+RC0_S-P_^2^+RC45_S-P_^2^)^0.5^, (TRSEQ_S-P_^2^+TRC0_S-P_^2^+TRC45_S-P_^2^)^0.5^, and (BRSEQ_S-P_^2^+BRC0_S-P_^2^+BRC45_S-P_^2^)^0.5^, respectively). Considering the 90% confidence interval of the lateral differences of the biometric measures, the average corneal front and back surface power (RSEQ and BRSEQ) are within limits of -0.64 to 0.63 dpt and -0.12 and 0.12 dpt, AL is within-0.45 and 0.43 mm, CCT within -0.01 and 0.01 mm, ACD within -0.20 and 0.20 mm, and LT within -0.13 and 0.14.

**Fig 3:**
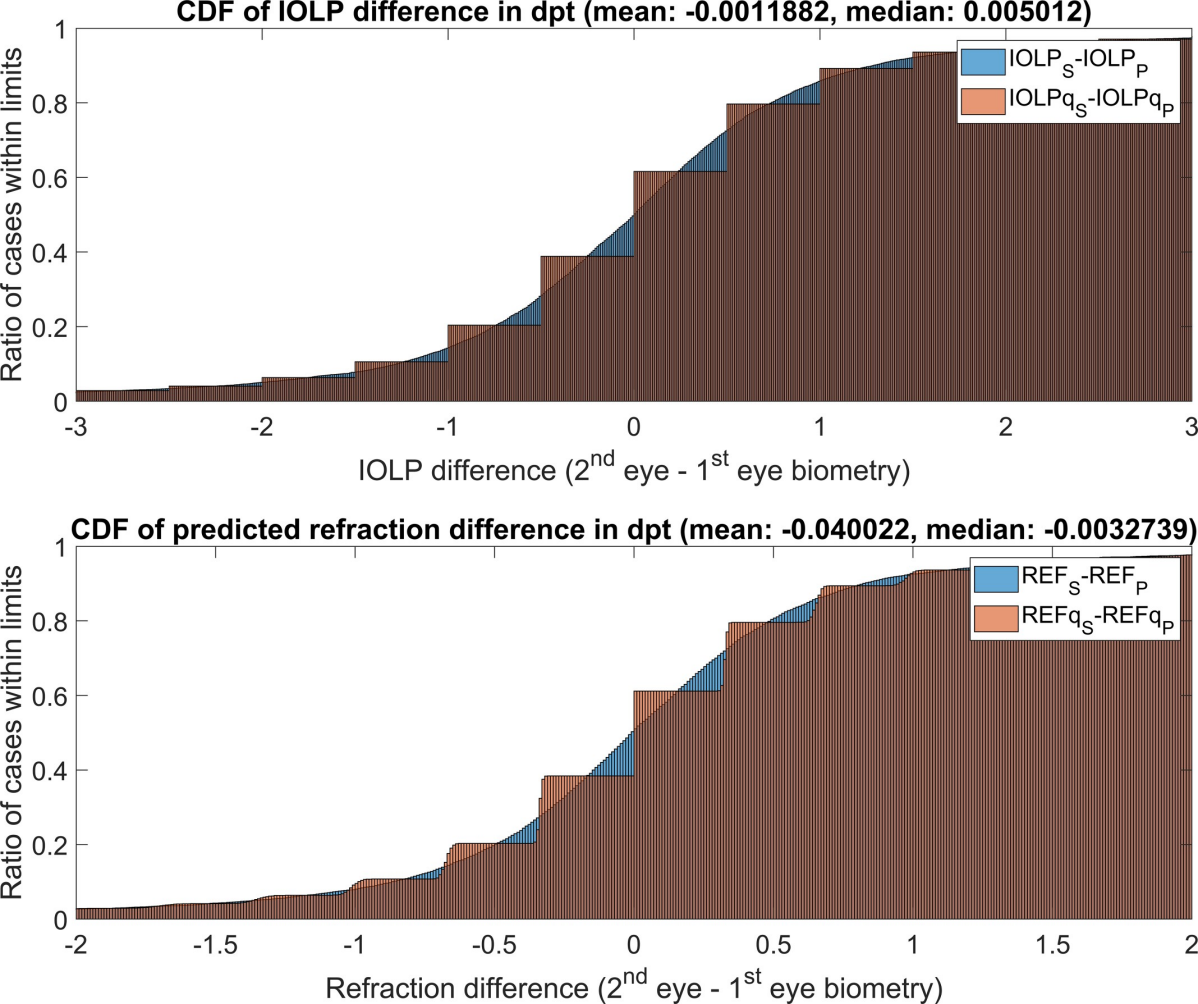
Normalised cumulative density functions (CDF): In the upper graph the difference between the lens power derived with the biometry of the secondary eye and the lens power derived with the biometry of the primary eye is shown (IOLP_S-P_ = IOLP_S_-IOLP_P_), whereas in the lower graph the resulting predicted refraction at the spectacle plane (REF_S-P_ = REF_S_-REF_P_) is displayed where the intraocular lens derived from the secondary eye biometry (IOLP_S_) is inserted (instead of IOLP_P_) in the primary eye. The overlay (in red) displays the ‘real world’ scenario of lenses available in steps (here: Quantised to half dioptre steps) and the lens power IOLPq_S-P_ = IOLPq_S_-IOLPq_P_ (upper graph). The respective refractive outcome is provided in the lower graph (REFq_S-P_ = REFq_S_-REFq_P_).

Using the Castrop lens power calculation formula [13], the lens power was calculated using the biometry from the primary eyes (IOLP_P_) and also using the biometry from the secondary eyes (IOLP_S_). The cumulative density function of the differences IOLP_S-P_ = IOLP_S_-IOLP_P_ is shown in **Fig 3** in the upper graph in blue.

The cumulative density function for the predicted difference in refraction at the spectacle plane where a lens with the power IOLP_S_ is inserted in the primary eye (instead of IOLP_P_) is shown on the lower graph (in blue). In addition, as lenses are only available with specific power steps, the difference of the quantised lens power derived from the secondary eye biometry (IOLPq_S_) and the primary eye biometry (IOLPq_P_) is provided (in red) in the upper graph. The predicted refraction at the spectacle plane (REFq_S-P_) where a lens with a power IOLPq_S_ is inserted in the primary eye (instead of IOLPq_P_) is shown in the lower graph (in red). In this dataset, if a lens with the power IOLP_S_ were inserted in the primary eye, 34.54% / 60.65% / 84.63% / 95.02% of eyes would end up within REF_S-P_ = ±0.25 dpt / ±0.50 dpt / ±1.00 dpt / ±2.00 dpt of refractive error. The respective percentages for REFq_S_P_ within limits yielded 22.76% / 59.32% / 83.60% / 94.91%.

On the left side of **Table 3,** the descriptive data of the lens power calculated with the Castrop formula for emmetropia based on the primary eye biometry or the secondary eye biometry (IOLP_S_) are displayed, together with the difference IOLP_S-P_, and the difference between the quantised lens powers IOLPq_S-P_. On the right side, the descriptive data for predicted refraction at the spectacle plane REF_P_ / REF_S_ / REFq_P_ and REFq_S_ are shown where the lens power is derived from the primary and secondary eye biometry, or from the quantised lens power from the primary and secondary eye biometry. Considering the 90% confidence interval of the intraocular lens power lateral differences, the IOLP_S-P_ is within limits of -2.00 and 2.02 dpt and IOLPq_S-P_ within -2.00 and 2.00 dpt. The respective confidence interval for the predicted refraction lateral differences at the spectacle plane ranges within -1.36 to 1.30 dpt (REF_S-P_) if the non-quantised lens is considered and within -1.36 to 1.31 dpt (REFq_S-P_) if the quantised lens is considered.

**Table 3:** Left side: Descriptive data of the lens power calculated with the Castrop formula for emmetropia based on the primary eye biometry (IOLP_P_) or the secondary eye biometry (IOLP_S_), difference IOLP_S_-IOLP_P_ (IOLP_S-P_), and the difference between lens powers IOLPq_S_-IOLPq_P_ (IOLPq_S-P_) where both lenses are quantised in half dioptres steps. Right side: Predicted refraction at the spectacle plane if IOLP_P_ (as the perfect lens, refraction equals zero) would be inserted (REF_P_), if IOLP_S_ would be inserted (REF_S_), together with the difference REF_S-P_ = REF_S_-REF_P_ and the difference REFq_S-P_ = REFq_S_-REFq_P_ for the ‘realistic case’ where the quantised lens IOLPq_S_ derived from the secondary eye biometry is inserted instead of the quantised lens (IOLPq_P_) derived from the primary eye biometry.

	IOLP_P_	IOLP_S_	IOLP_S-P_	IOLPq_S-P_	REF_P_	REF_S_	REF_S-P_	REFq_S-P_
	Intraocular lens power in dpt				Predicted refraction in dpt			
MEAN	20.37	20.36	0.00	0.00	0.00	-0.04	-0.04	-0.04
SD	4.33	4.36	1.96	1.97	0.00	2.72	2.72	2.74
MEDIAN	20.93	20.92	0.00	0.00	0.00	0.00	0.00	0.00
5% quantile	12.64	12.39	-2.00	-2.00	0.00	-1.36	-1.36	-1.36
95% quantile	26.13	26.14	2.02	2.00	0.00	1.30	1.30	1.31
0.5% quantile	3.09	3.30	-7.03	-7.00	0.00	-4.78	-4.78	-4.89
99.5% quantile	31.77	32.05	6.81	7.00	0.00	4.35	4.35	4.41

MEAN, SD, and MEDIAN refer to the mean value, standard deviation and median value, and 5% / 9% quantiles and 0.5% / 99.5% quantiles to the 90% and 99% confidence interval, respectively.

## Discussion

In clinical situations where a biometric measurement of one eye of an individual is not available prior to surgery, ophthalmic surgeons use the corresponding measurement from the biometry of the fellow eye [1,2,6,16]. This can happen especially in cases of cataract surgery with a very opaque crystalline lens (where optical biometers fail); in situations without stable fixation; or with gas or silicone oil replacing the native vitreous (which will be replaced later on). Also, the surgeon may use the data of the fellow eye instead of ultrasound biometry which is known to be much less accurate [1].

Only limited data on the similarity of eyes in the context of ocular biometry prior to cataract surgery are available in the literature [7,8,16–18]. Similarly, data on anisometropia of anterior segment dimensions are only sparingly documented [19,20]. In our study we used a very large dataset from a modern optical biometer, filtered for complete data (all parameters indexed by the biometer with ‘OK’) for the left and the right eye of individuals. This dataset was split randomly such that one eye of each individual was indexed as primary and the other as secondary.

Classical lens power calculation concepts such as the SRKT, Hoffer-Q or Holladay1 formula use only the axial length and the K value as a measure of the corneal front surface curvature. These are transferred to refractive power using the Javal keratometer index, whereas CCT and the corneal back surface curvature are ignored. Enhanced lens power calculations include more biometric data, in order to reduce the predicted refraction error, defined as the difference between the achieved refraction (spherical equivalent) and the intended refraction. Some concepts use the anterior chamber depth, as this parameter was measurable with the first generation of optical biometers, whereas others also include data on the central thickness of the crystalline lens [1], the horizontal corneal diameter, central corneal thickness, the corneal back surface curvature, the sex or the age of the patient [13,15].

In the present study we analysed the differences in all relevant biometric measures as provided by a modern optical biometer. The data of the secondary eye and the primary eye were compared in order to get an insight into the amount of variation between the measures of both eyes of an individual. As the axes of the principal meridians of corneal power do not generally match between both eyes, the radii and axis orientations cannot be directly subtracted. Instead, we had to perform a decomposition of the radii of curvature in both meridians and of the orientation into power vectors, in order to end up with 3 vector components. These could then be directly subtracted from the respective values of the fellow eye in order to read out the lateral differences. These vector components in terms of spherical equivalent power and projection of the astigmatism to the 0°/90° meridian and the 45°/135° meridian calculated for the corneal front surface, total keratometry, and corneal back surface, encompass identical information as in the classical notation of ophthalmologists with both refractive powers in the principal meridians including the axis orientation, but they facilitate statistical analysis.

From the IOLMaster 700 dataset we included the corneal front surface curvature data, total keratometry as a measure for the cornea considered as a thick lens with front and back surface, together with corneal back surface curvature. Keratometry, total keratometry and back surface curvature were transferred to power vector components for a direct comparison between both eyes of an individual. To account for a potential symmetry of corneal astigmatism with respect to the vertical axis, we calculated an additional power vector component for keratometry (RC45OD), total keratometry (TRC45OD) and the corneal back surface (BRC45OD) where the respective component of the left eyes were flipped in sign to simulate right eye situations only. From **Fig 1** and **Table 1** we can directly read out that the corneal front surface shows, on average, a trend towards astigmatism with the rule (positive values for RC0 for primary and secondary eyes) whereas the corneal back surface shows, on average, a trend towards astigmatism against the rule (negative values for BRC0 for primary and secondary eyes). This means that the corneal front surface astigmatism with the rule e.g. measured with a keratometer (on average 0.20 dpt) is mostly compensated by the back surface astigmatism against the rule (on average -0.24 dpt) in such a way that the astigmatism of total keratometry is around zero (on average -0.01 dpt).

In addition, we included the central corneal thickness; the (external) anterior chamber depth, defined as the distance from the anterior corneal apex to the lens front apex; the central thickness of the crystalline lens, and the horizontal corneal diameter. The lateral differences in these parameters are listed in **Table 2**. The corneal back surface measures are ignored In most lens power calculation concepts. This means that we assume a fixed ratio of front to back surface curvature, as used with all manual keratometers, automatic keratometers (e.g. integrated in an optical biometer) or topographes. However, the validity of this assumption cannot be verified without measurement of corneal back surface curvature.

To assess the impact of the lateral differences in the biometric measures on the intraocular lens power or the predicted refraction after implantation of a lens, as calculated from the biometric data of the fellow eye, we implemented the Castrop formula [13,15] as a newest generation paraxial lens power calculation concept, based on a thick lens cornea model and a prediction algorithm for the effective lens position based on the axial length, the anterior chamber depth and phakic lens thickness and 2 formula constants (C and H). This lens power calculation formula considers both the CCT and the curvature of the corneal front and back surfaces. Without loss of generality, formula constants were taken from the IOLCon WEB site (https://iolcon.org) for the Tecnis lens (Johnson & Johnson Vision). For all primary and secondary eyes we calculated the respective lens power for postoperative emmetropia, and the difference of lens power from the secondary eye and the primary eye was analysed. As lenses are generally available in power steps (mostly in half dioptre steps in the central delivery range), we also quantised the lens power for the primary and secondary eye to create more realistic conditions. This quantisation was performed asymmetrically according to typical clinical practice, meaning that to avoid postoperative hypermetropia 0.15 dioptre was added to the lens power before rounding to half dioptre steps. Then, in a last step, the lens powers from the primary eye, the secondary eye, and the quantised lens power for the primary and secondary eye were used to predict the postoperative refraction of the primary eye at the spectacle plane. The respective graphs with the cumulative density function for the difference in lens power based on the secondary and primary eye biometry and the resulting refraction are shown in **Fig 3**.

At this point we would like to provide 3 clinical examples from our dataset to show the effect of anisometropia: In example 1 the right eye (P) biometric data (AL: 24.59 mm, RF: 8.02 mm, RB: 7.12 mm, CCT: 517 μm, ACD: 3.66 mm, LT: 4.31 mm) of a 70 year old lady were used to predict the lens power of 19.51 dpt (19.5 dpt lens is implanted). The respective biometric data from the left eye were AL: 25.20 mm, RF: 7.95 mm, RB: 7.15 mm, CCT: 512 μm, ACD: 3.70 mm, LT: 4.31 mm, and the resulting lens power is 16.94 dpt (17.0 dpt lens would be appropriate). If a 19.5 dpt lens were implanted in (S) instead of a 17.0 dpt lens, the refraction after cataract surgery is predicted to be -1.72 dpt instead of -0.04 dpt. In example 2 the left eye (P) biometric data (AL: 22.85 mm, RF: 7.58 mm, RB: 6.89 mm, CCT: 566 μm, ACD: 3.05 mm, LT: 4.61 mm) of a 74 year old lady were used to predict the lens power of 21.57 dpt (21.5 dpt lens is implanted). The respective biometric data from the left eye were AL: 25.20 mm, RF: 7.64 mm, RB: 6.97 mm, CCT: 562 μm, ACD: 2.82 mm, LT: 4.78 mm, and the resulting lens power is 22.73 dpt (23.0 dpt lens would be appropriate). If a 21.5 dpt lens were implanted in (S) instead of a 23.0 dpt lens, the refraction after cataract surgery is predicted to be 0.81 dpt instead of -0.18 dpt. In example 3 the right eye (P) biometric data (AL: 23.56 mm, RF: 7.71 mm, RB: 6.99 mm, CCT: 554 μm, ACD: 3.13 mm, LT: 4.65 mm) of an 80 year old man were used to predict the lens power of 20.39 dpt (20.5 dpt lens is implanted). The respective biometric data from the left eye were AL: 22.26 mm, RF: 7.86 mm, RB: 7.17 mm, CCT: 564 μm, ACD: 3.04 mm, LT: 4.63 mm, and the resulting lens power is 26.64 dpt (27.0 dpt lens would be appropriate). If a 20.5 dpt lens were implanted in (S) instead of a 27.0 dpt lens, the refraction after cataract surgery is predicted to be 3.85 dpt instead of -0.24 dpt. In the first two of these examples, the outcome would have been suboptimal but moderate, whereas in the third example, the consequences of using the contralateral biometry would have been more severe.

**Fig 4:**
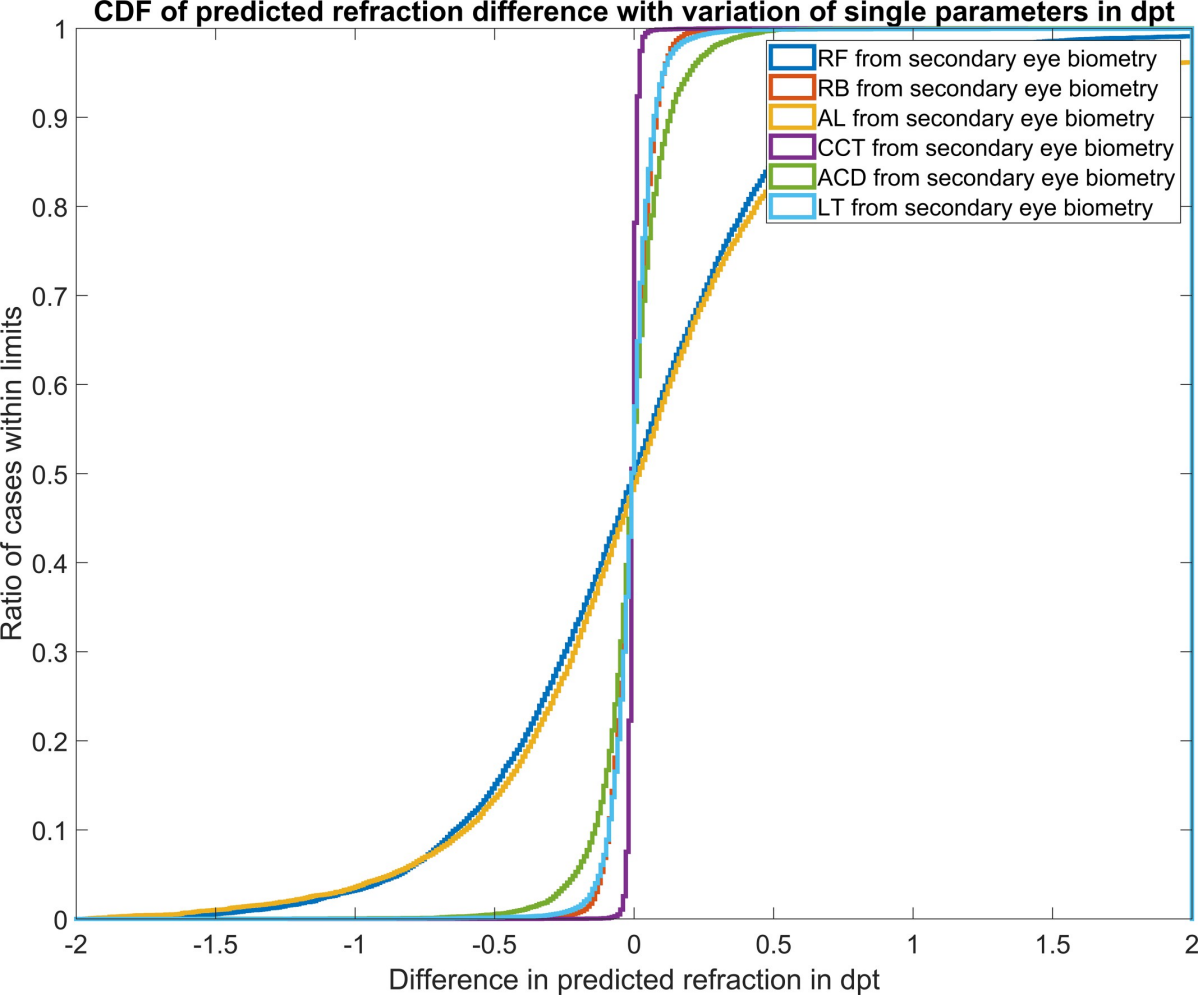
Cumulative density function (CDF) for the predicted refraction if–instead of using *all* parameters from the fellow eye–*only one* biometric parameter from the fellow eye (secondary eye) is used to calculate the intraocular lens power. From this lens power we back-traced the predicted refraction at the spectacle plane. RF / RB / AL / CCT / ACD / LT from secondary eye biometry refers to the situation where only the corneal front surface curvature /corneal back surface curvature / axial length / central corneal thickness / anterior chamber depth / the thickness of the crystalline lens is replaced in the lens power calculation by the respective value from the secondary eye.

To go into even more detail, we aimed to identify the effect of each potential biometric effect size on the predicted refraction. Therefore, we used the biometric data of the primary eye for calculation of the intraocular lens power and analysed the effect if *only one* of the measures were replaced by the respective biometric value of the secondary eye. This strategy was performed for the corneal front surface curvature RF, the corneal back surface curvature RB, the axial length AL, central corneal thickness CCT, anterior chamber depth ACD as well as thickness of the crystalline lens LT. The respective cumulative density functions are shown in an overlay in **Fig 4**.

From this plot we directly see that the dominant effect sizes are the AL and the corneal front surface curvature, followed by the ACD, LT and corneal back surface curvature which have a minor effect and CCT which has no significant impact if taken from the secondary eye instead of the primary eye. In particular, the use of the anterior chamber depth from the fellow eye could be of major clinical relevance in situations where the primary eye is already pseudophakic and a replacement of the lens (with unknown power) is necessary, and the fellow eye is still phakic.

In **conclusion**, in some situations where biometry cannot be performed prior to cataract surgery, the use of the biometry of the fellow eye yields sufficient results for the lens power calculations and for the prediction of the refractive outcome, as a result of the strong similarity of the biometric measures of both eyes. The most critical measures are the axial length and the corneal front surface curvature, whereas the anterior chamber depth, the lens thickness and the corneal back surface curvature have only a minor effect on the refractive result.
